# Externalizing personality characteristics define clinically relevant subgroups of alcohol use disorder

**DOI:** 10.1371/journal.pone.0265577

**Published:** 2022-03-18

**Authors:** Ildikó Kovács, Bernadett I. Gál, Zsolt Horváth, Ildikó Demeter, Sándor Rózsa, Zoltán Janka, Róbert Urbán, Zsolt Demetrovics, Bálint Andó

**Affiliations:** 1 Department of Psychiatry, Albert Szent-Györgyi Medical School, University of Szeged, Szeged, Hungary; 2 Doctoral School of Psychology, ELTE Eötvös Loránd University, Budapest, Hungary; 3 Institute of Psychology, ELTE Eötvös Loránd University, Budapest, Hungary; 4 Department of Psychiatry, Washington University School of Medicine, St. Louis, MO, United States of America; 5 Institute of Psychology, Károli Gáspár University of the Reformed Church in Hungary, Budapest, Hungary; 6 Centre of Excellence in Responsible Gaming, University of Gibraltar, Gibraltar, Gibraltar; Washington University, St. Louis, UNITED STATES

## Abstract

**Aims:**

Higher levels of externalizing characteristics, i.e. impulsivity, novelty seeking and aggression, could contribute to the development, progression and severity of alcohol use disorder (AUD). The present study aims to explore whether these externalizing characteristics together have a potential group-forming role in AUD using latent profile analysis (LPA).

**Methods:**

Externalizing characteristics of 102 AUD patients were analyzed using LPA to explore the group-forming role of externalizing symptoms; groups were compared in terms of demographic and alcohol-related variables, indices of psychopathological, depressive and anxiety symptom severity.

**Results:**

LPA revealed and supported a two-group model based on externalizing symptoms. The group with higher levels of externalizing symptoms showed significantly elevated levels of alcohol-related and anxio-depressive symptoms.

**Conclusions:**

Externalizing characteristics converge and have a group-forming role in chronic AUD, and are associated with a more severe form of AUD. By making the diagnostic category less heterogeneous, these different subtypes within AUD may provide aid in tailoring treatments to patients’ specific needs.

## Introduction

Alcohol use disorder (AUD) has an immense health-related, socio-cultural and economic burden [1], which underscore the need for the in-depth understanding of the symptomatic patterns related to the disease. Scientific literature thoroughly describes externalizing symptom characteristics, i.e. impulsivity, novelty seeking and aggression, that accompany AUD from the development through its progression to relapse or recovery [2]; and these features may also contribute to its high comorbidity with the most common neuropsychiatric disorders like other impulse control and anxio-depressive disorders [3,4]. Therefore, further comprehensive examination of externalizing characteristics is essential in case of AUD, since these characteristics could be more pronounced in specific AUD subgroups [5], and could not only be presented as vulnerability markers, but could be associated with more severe forms of the disorder [2,6–8]. Moreover, the increased level of novelty seeking contributes to the higher risk of lower therapeutic compliance and earlier dropouts [9]. Aggression also plays an important role in mediating the severity of alcohol use [10], and its connection with problem drinking is well-documented [11,12]. Together, the joint role of externalizing symptoms in AUD could play a pivotal role not just at the beginning of maladaptive alcohol use, but in the maintenance of the disease and during relapses that may accompany patient recovery [7,13], and they could be associated with other concomitant features like the elevated level of anxiety or the emergence of depressive symptoms [14]. Scientific literature widely addresses the comorbidity of anxio-depressive symptoms with AUD [4], which can also occur in distinct subgroups of the disorder [15–17]. However, it has still remained unclear whether externalizing symptoms have a group-forming role and what specific pattern they present with regard to anxio-depressive symptom co-occurrence.

It is documented that these externalizing markers separately have paramount importance in AUD, but the question arises whether these characteristics converge, and jointly aid in deconstructing the heterogeneous diagnostic category in AUD. We hypothesize that externalizing characteristics have a group-forming role in AUD, and patients with more expressed externalizing symptoms are associated with more severe anxio-depressive symptoms.

## Materials and methods

### Procedure and participants

As part of a comprehensive research project, patients with chronic AUD receiving inpatient treatment were involved from the Department of Psychiatry, Faculty of Medicine, University of Szeged. The inclusion criteria for participation in this study were the following: having met the criteria of DSM-5 diagnosis of AUD, completing at least primary education, surpassing the level of intellectual disability (IQ above 70). Participants were excluded if they had past history of comorbid substance use disorder (except caffeine and/or nicotine), neurodegenerative or neurological disorders or psychosis spectrum disorders. For a detailed description of exclusion and inclusion criteria and patient enrolment, see Kovács et al. [3,18]. Data of 102 patients with AUD were analyzed (Mean age: 45.68, SD: 10.35, 71.2% men, age of onset: regular alcohol consumption: 24.61 SD: 10.09, Education% (primary: 7.8%; secondary 70.9%; higher 21.4%)). The study was conducted in accordance with the Declaration of Helsinki and was approved by the Human Investigation Review Board, University of Szeged (ethical approval number: 49/B-53/2016KK). Prior to enrolment, every patient signed an informed consent form.

### Measures

Data were collected for all patients after the withdrawal syndrome has subsided. Addiction Severity Index semi-structured clinical interview was applied to reveal the following (AUD-related) demographic data: age, gender, education, age of onset of regular alcohol use, family history of AUD, and number of previous inpatient treatments [19]. The severity of AUD was assessed with the 20-item self-administered questionnaire, the Severity of Alcohol Dependence Questionnaire, which measures the psycho-biological aspects of alcohol withdrawal [20]. The following self-measurement scales were applied to operationalize externalizing characteristics: The 29-item Buss-Perry Aggression Questionnaire (BPAQ) was used to reveal four facets of physical and verbal aggression, hostility and anger [21,22]. The 21-item Barratt Impulsivity Scale (BIS) was calculated to assess different components of impulsiveness, such as cognitive impulsivity, behavioral impulsivity and impatience & restlessness [23,24]. The individual level of Novelty-seeking was measured with the Temperament and Character Inventory–Revised (TCI-R), which is one of the most widely used tool developed to measure personality traits assessing four temperament and three character dimensions, from which novelty seeking is a temperament dimension [25,26]. The anxio-depressive characteristics were measured by the total scores of the 21-item Beck Depression Inventory, which evaluated the severity of depressive symptoms [27]; and the 40-item Spielberger State–Trait Anxiety Inventory [28,29] for measuring the presence of state and trait anxiety.

### Statistical analysis

To test the hypothesis whether externalizing characteristics have a group-forming role in AUD, latent profile analysis (LPA) was conducted. This method can allow to identify empirically-based groups with distinct profiles of externalizing characteristics. That is, LPA can contribute to having more precise classification models than other, more arbitrary classification methods (e.g., splitting participants into categories based on median, and/or quartiles). The present analytical approach was in line with previous latent class analytic studies which examined the link between problematic alcohol use and psychopathological symptoms [30].

The estimated models contained three continuous and observed indicator variables: aggression, novelty seeking and impulsivity. All variables were standardized to assist interpretation. These constructs were measured by the BPAQ total score, by the novelty seeking total score of the TCI-R and by the BIS total score, respectively. Previous studies also calculated total scores based on these questionnaires to assess these externalizing constructs [3,31]. Moreover, the use of the total scale scores was also warranted due to the relatively low sample size (instead of the use of the more specific subscale scores). Independence of the indicator variables was suggested by significant, positive and moderately strong correlations between aggression, novelty seeking and impulsivity (r = 0.34–0.47; S1 Table). Previous theoretical and empirical findings also supported the distinguishability of these constructs (see: Introduction).

LPA models with increasing number of latent classes were estimated starting with the most parsimonious model with one latent class, and were compared in terms of various model fit indices. Lower levels of the Akaike and the Bayesian Information Criteria (AIC and BIC) and the sample size-adjusted Bayesian Information Criteria (SSA-BIC), and higher level of classification accuracy based on the measure of Entropy indicated more optimal model fitness. Decision regarding the number of classes to be retained was primarily based on the Lo-Mendel-Rubin adjusted likelihood ratio test (LMR-LRT): it tested whether a given model with k number of latent classes offered a more optimal and parsimonious solution in contrast with a model with k-1 number of latent classes. The maximum likelihood robust to non-normality (MLR) estimation method was used to perform LPA.

To test the study hypothesis whether AUD patients with more expressed externalizing symptoms show more severe anxio-depressive symptoms, the latent classes were compared in terms of trait and state anxiety and depressive symptoms. Furthermore, the identified latent classes were also compared in terms of socio-demographic and alcohol misuse related variables. For the continuous validating variables (i.e., age, age of onset of regular alcohol consumption, number of previous inpatient treatments, alcohol dependence severity, depressive symptom severity, state and trait anxiety), the 3-step Block-Croon-Hagenaars (BCH) method was used to perform bivariate comparisons between the latent classes [32]. Chi-square statistic (χ^2^) was calculated as a test statistic related to the BCH method [32], and Cohen’s d represented effect size. For the comparisons in terms of categorical validating variables (i.e., gender, level of education, family history of AUD), Pearson’s Chi-square statistic (χ^2^) was calculated as a test statistic with Fisher’s exact test for significance testing. Effect sizes were represented by Phi correlation estimates (φ).

Furthermore, to control for potential confounding effects, a multivariate logistic regression model was also constructed by using the R3Step method [33]. This allowed to compare the latent classes in terms of anxio-depressive symptoms while controlling for socio-demographic and alcohol misuse related variables. As there were very high correlations between depressive symptoms, trait and state anxiety (r = 0.69–0.77; S1 Table), a composite score of anxio-depressive symptoms was constructed by using principal component analysis (component weights = 0.87–0.92; explained variance = 82.06%). The latter step was necessary to avoid issues related to multicollinearity in the multivariate model. Other validating variables did not present high correlations with each other (S1 Table) [34].

Analyses were carried out by using Mplus 8.0 [35] and IBM SPSS Statistics 26.0 software [36].

## Results

### Model selection

Models with one to four latent classes were estimated and assessed (see Table 1). Based on the indices of the AIC, the SSA-BIC and the Entropy, the four-class model provided the most optimal model fit. However, the BIC and the LMR-LRT indices suggested that the two-class solution showed the most optimal classification. The LMR-LRT showed non-significant result for both the three-class and the four-class models, which indicated that it might not be reasonable to include an additional latent class over two groups.

**Table 1:** Model fit indices for models with different number of latent classes (N = 102).

	AIC	BIC	SSA-BIC	Entropy	LMR-LRT	p
1-class model	880.39	896.14	877.19	-	-	-
2-class model	846.78	873.03	841.44	0.67	39.48	0.008
3-class model	839.77	876.52	832.30	0.79	14.24	0.111
4-class model	836.32	883.57	826.71	0.85	10.87	0.056

Note. AIC = Akaike Information Criteria; BIC = Bayesian Information Criteria; SSA-BIC = Sample size adjusted Bayesian Information Criteria; LMR-LRT = Lo-Mendel-Rubin adjusted likelihood ratio test.

Due to these conflicting findings, it was not possible to determine unequivocally the best fitting model. Overall, due to multiple considerations, the two-class model was selected as the most optimal classification solution and retained for further analyses. First, previous studies suggested that results from the LMR-LRT can indicate the number of classes to be retained more precisely compared with the other fit indices [37]. Second, in the three-class and the four-class models only one and two participants were assigned to some of the latent classes. Thus, LPA was performed again without these three potential outlier cases. The model fit indices suggested similar pattern than in the full sample (S2 Table). The indices of the AIC, the SSA-BIC and the Entropy indicated that the four-class model presents the most optimal model fit. Alternatively, the BIC and the LMR-LRT suggested that the two-class solution should be retained. The latent classes of the two-class model presented similar characteristics to the full sample, thus it was possible to replicate these classes even after the exclusion of the potential outlier cases. However, in the cases of the three-class and the four-class models, the best loglikelihood values were not replicated, therefore these solutions might not have been trustworthy. Therefore, the two-class model was considered as the most reliable solution out of the competing models. Finally, it was also an aim to select a more parsimonious solution (i.e., a model with fewer latent classes) as it was not possible to determine unequivocally the best fitting model.

### Profile characteristics of the latent classes

**Fig 1:**
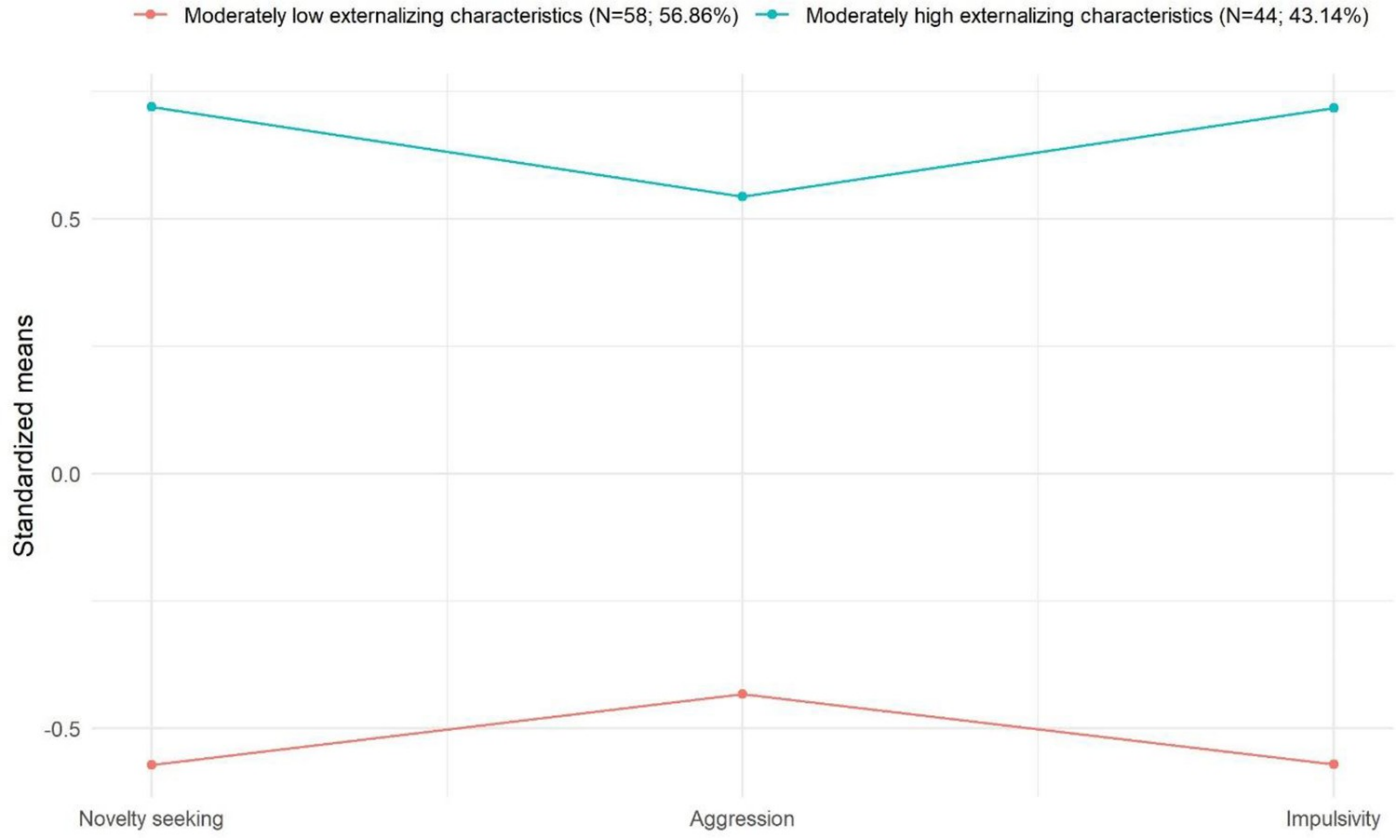
Profile characteristics of the latent classes. All indicator variables are standardized in order to facilitate understanding of class-based characteristics. Therefore, each indicator variable’s mean equals 0 and standard deviations equal 1.

To examine profile characteristics, class-based mean scores of the indicator variables were considered. Fig 1 displays the profile characteristics of the latent classes. Class 1 (‘Moderately low externalizing characteristics’) presented moderately low rates of novelty seeking, aggression and impulsivity. In the case of Class 2 (‘Moderately high externalizing characteristics’), moderately high levels of novelty seeking, aggression and impulsivity were shown. The average latent class probabilities for the most likely latent class memberships were 0.91 and 0.91, respectively.

### Associations of the latent classes with anxio-depressive symptoms and demographic variables

Results of the latent class comparisons are presented in Table 2. Members of Class 2 showed significantly higher levels of severity of alcohol dependence, depressive symptoms, and levels of trait and state anxiety compared to Class 1 with strong effect sizes in each case. Class 2 was also characterized by significantly lower age of onset of regular alcohol drinking (with moderate effect size) and by higher rates of lack of vocational or high-school graduation and family history of AUD (with small effect sizes).

**Table 2:** Comparison of latent classes in terms of demographic and anxio-depressive variables.

	Class 1 ‘Moderately low externalizing characteristics’ N = 58 (56.86%)	Class 2 ‘Moderately high externalizing characteristics’ N = 44 (43.13%)	χ^2^ (p)	Effect size
Male gender^1^ N (%)	45 (77.59%)	33 (75.00%)	0.09 (0.816)	φ = 0.03
Age M (S.E.)	47.69 (1.44)	43.61 (1.72)	2.77 (0.096)	d = 0.37
Level of education^2^: lack of vocational or high-school graduations N (%)	41 (70.69%)	39 (88.64%)	4.76 (0.032)	φ = 0.22
Family history of alcohol use disorder^3^ N (%)	41 (70.69%)	39 (88.64%)	4.76 (0.032)	φ = 0.22
Age of onset: regular alcohol consumption M (S.E.)	27.24 (1.57)	21.21 (1.40)	6.94 (0.008)	d = 0.55
Number of previous inpatient treatments M (S.E.)	3.02 (0.62)	3.55 (1.24)	0.12 (0.729)	d = 0.08
Severity of alcohol dependence M (S.E.)	21.49 (1.74)	35.29 (1.64)	28.65 (<0.001)	d = 1.12
Depressive symptoms M (S.E.)	9.45 (1.07)	20.46 (1.84)	22.96 (<0.001)	d = 1.09
State anxiety M (S.E.)	43.41 (1.27)	54.23 (1.78)	20.81 (<0.001)	d = 0.94
Trait anxiety M (S.E.)	37.87 (1.58)	49.01 (1.77)	18.78 (<0.001)	d = 0.94

Note. For continuous variables each cell shows mean (M) and standard error (S.E.) values, Chi-square (χ^2^) test statistics are calculated based on the Block-Croon-Hagenaars (BCH) method, and Cohen’s d represents effect size. For categorical variables, number of subjects (N) and their within-class proportion (%) for a given category are shown in each cell, Chi-square (χ^2^) test statistics (with Fisher’s exact test for significance test) represents the overall differences between the groups, and Phi correlation estimates (φ) show the effect size.^1^Gender: reference category = Females. ^2^Level of education: reference category = vocational graduation. ^3^Family history of alcohol use disorder: reference category = no history of alcohol use disorder in the participant’s family.

Next, binary logistic regression was performed to control for potential confounding effects in the comparisons. Table 3 presents predictive effects on the membership of the ‘Moderately high externalizing characteristics’ latent class. Due to the small sample size and the limited statistical power to detect significant effects, only those variables were included in the multivariate model that showed significant differences between the two classes (Table 2). Higher levels of alcohol dependence severity and anxio-depressive symptoms had significant and positive predictive effects on the membership of the ‘Moderately high externalizing characteristics’ latent class.

**Table 3:** Binary logistic regression: Predictive effects on the membership of the ‘Moderately high externalizing characteristics’ latent class.

	OR	p
Level of education^1^: lack of vocational or high-school graduations	2.50	0.355
Family history of alcohol use disorder^2^	6.88	0.115
Age of onset: regular alcohol consumption	0.94	0.190
Severity of alcohol dependence	1.13	0.031
Anxio-depressive symptoms	4.72	0.025

Note. Reference category: ‘Moderately low externalizing characteristics’, N = 58 (56.86%). OR: odds ratio.

^1^Level of education: reference category = vocational graduation.

^2^Family history of alcohol use disorder: reference category = no history of alcohol use disorder in the participant’s family.

## Discussion

Our study investigated externalizing characteristics that individually have pivotal roles in addiction research, but this is the first examination to reveal that externalizing symptoms together converge and have a clinically significant group-forming role among patients with chronic AUD leading to two, well-distinguishable groups. The group with higher levels compared to the group with lower levels of externalizing characteristics showed elevated levels of alcohol consumption and anxio-depressive symptoms, which indicated a distinct and more severe symptom profile, thus a more severe form of addiction. While earlier typological models detected externalizing and anxio-depressive symptoms as hallmarks of separate subpopulations [15–17], our study identified that these could co-occur. This finding is also in line with the results of Horváth et al. [30] who detected that a subgroup of alcohol users in a community sample is characterized by both increased externalizing and anxio-depressive symptoms. Based on our results, externalizing symptom characteristics not just have a group-forming role helping in disentangling the heterogeneity of the diagnostic category of AUD, but is suitable for detecting subgroups characterized with concomitant anxio-depressive features, thus a more severe subgroup of AUD.

The relapsing, chronic nature of AUD and the heterogeneity within the diagnostic category [38] cause enormous challenge for the treatment of AUD in general [39]. Personalized treatment planning of chronic AUD is of supreme importance in the selection of optimal continuing care interventions [40]. The different subtypes within the chronic AUD group may influence patients’ attitudes to seek help and the planning of personalized therapeutic approaches as well [17]. Successful relapse prevention and complete abstinence could seem an unattainable treatment aim in severe, chronic AUD [41,42]. For example, evidence suggests that AUD groups characterized by increased externalizing symptoms seek less therapeutic help [17]. Based on our study, the group-forming role of these characteristics deserves more attention, hence empirical subtypes could further help rehabilitation programs to develop more personalized therapeutic plans for a wide range of patients with chronic AUD.

The present study is unique in the literature, since group formation was performed along externalizing characteristics that are individually proven to be key in AUD, but haven’t been evaluated jointly; while demographic and anxio-depressive symptom markers were used only for post-confirmation. However, the conclusions drawn could only be generalized to a limited extent, due to the modest sample size, and concerning the cross-sectional nature of this study, no causal consequences could be drawn. It is also important to consider that it was not possible to determine unequivocally the best fitting latent class model. Despite these limitations, the present study suggests that the different subtypes within chronic AUD could contribute to making the diagnostic category less heterogeneous, and may deserve more attention, since empirical subtypes could further help rehabilitation programs to develop more personalized therapeutic approaches.

## Statements

### Statements of ethics

The study was conducted in accordance with the Declaration of Helsinki and was approved by the Human Investigation Review Board, University of Szeged (ethical approval number: 49/B-53/2016KK). Prior to enrolment, every patient signed an informed consent form.

## Supporting information

S1 Table Bivariate correlations between the variables. Notes. N = 100. Values in the table are Pearson’s correlation estimates (r) and robust bootstrap-based, bias-corrected, accelerated 95% confidence intervals (95% BCa CI). Level of significance: *p<0.050; **p<0.010; ***p<0.001. 1Coded as: 0 = Female, 1 = Male. 2Coded as: 0 = Vocational graduation, 1 = Lack of vocational or high-school graduation. 3Coded as: 0 = No family history, 1 = Presence of family history. 4Composite Principal Component Analysis score based on the variables measuring depressive symptoms, state and trait anxiety.
(DOCX)

S2 Table Model fit indices of the different latent classes (N = 99). Note. AIC = Akaike Information Criteria; BIC = Bayesian Information Criteria; SSA-BIC = Sample size adjusted Bayesian Information Criteria; LMR-LRT = Lo-Mendel-Rubin adjusted likelihood ratio test. 1These solutions might not be trustworthy due to local maxima (i.e., the best loglikelihood value was not replicated).
(DOCX)
