# Supplementary material for: Externalizing personality characteristics define clinically relevant subgroups of alcohol use disorder
Source: PLoS One. 2022 Mar 18;17(3):e0265577. doi: 10.1371/journal.pone.0265577 (PMC8932598; doi:10.1371/journal.pone.0265577)
Supplement: S1 Table — Notes. N = 100. Values in the table are Pearson’s correlation estimates (r) and robust bootstrap-based, bias-corrected, accelerated 95% confidence intervals (95% BCa CI). Level of significance: *p<0.050; **p<0.010; ***p<0.001. 1Coded as: 0 = Female, 1 = Male. 2Coded as: 0 = Vocational graduation, 1 = Lack of vocational or high-school graduation. 3Coded as: 0 = No family history, 1 = Presence of family history. 4Composite Principal Component Analysis score based on the variables measuring depressive symptoms, state and trait anxiety. (DOCX) [file pone.0265577.s001.docx]

Supplementary Table 1. Bivariate correlations between the variables

|  | 1. | 2. | 3. | 4. | 5. | 6. | 7. | 8. | 9. | 10. | 11. | 12. | 13. |
| --- | --- | --- | --- | --- | --- | --- | --- | --- | --- | --- | --- | --- | --- |
| 1. Aggression | - |  |  |  |  |  |  |  |  |  |  |  |  |
| 2. Novelty seeking | 0.34***  [0.14; 0.51] | - |  |  |  |  |  |  |  |  |  |  |  |
| 3. Impulsivity | 0.40***  [0.18; 0.59] | 0.47***  [0.25; 0.64] | - |  |  |  |  |  |  |  |  |  |  |
| 4. Gender^1^ | -0.02  [-0.21; 0.16] | 0.03  [-0.17; 0.24] | -0.02  [-0.21; 0.17] | - |  |  |  |  |  |  |  |  |  |
| 5. Age | -0.04  [-0.22; 0.18] | -0.28**  [-0.46; -0.09] | -0.04  [-0.25; 0.16] | -0.09  [-0.26; 0.07] | - |  |  |  |  |  |  |  |  |
| 6. Level of education^2^ | 0.26**  [0.11; 0.40] | 0.26**  [0.08; 0.44] | 0.28**  [0.06; 0.49] | 0.11  [-0.11; 0.34] | -0.06  [-0.25; 0.10] | - |  |  |  |  |  |  |  |
| 7. Family history of alcohol use disorder^3^ | 0.17  [-0.04; 0.35] | 0.26*  [0.02; 0.45] | 0.19  [-0.06; 0.39] | -0.06  [-0.22; 0.13] | -0.24*  [-0.41; -0.06] | 0,22*  [0.00; 0.45] | - |  |  |  |  |  |  |
| 8. Age of onset: regular alcohol consumption | -0.28**  [-0.42; -0.11] | -0.36***  [-0.49; -0.21] | -0.17  [-0.37; 0.04] | -0.38***  [-0.57; -0.18] | 0.34**  [0.18; 0.50] | -0.38***  [-0.55; -0.18] | -0.24*  [-0.47; 0.02] | - |  |  |  |  |  |
| 9. Number of previous inpatient treatments | 0.09  [-0.11; 0.25] | 0.02  [-0.09; 0.19] | 0.07  [-0.10; 0.27] | 0.15  [0.05; 0.28] | 0.14  [-0.07; 0.27] | 0.14  [0.07; 0.27] | 0.11  [-0.02; 0.21] | -0.15  [-0.24; -0.10] | - |  |  |  |  |
| 10. Severity of alcohol dependence | 0.30**  [0.11; 0.45] | 0.41***  [0.26; 0.56] | 0.46***  [0.28; 0.62] | 0.08  [-0.12; 0.27] | -0.18  [-0.38; 0.03] | 0.15  [-0.07; 0.38] | 0.22*  [-0.01; 0.41] | -0.21*  [-0.40; -0.01] | 0.17  [0.06; 0.42] | - |  |  |  |
| 11. Depressive symptoms | 0.34***  [0.15; 0.53] | 0.19  [0.02; 0.35] | 0.54***  [0.38; 0.67] | -0.14  [-0.36; 0.10] | 0.09  [-0.09; 0.27] | 0.19  [-0.02; 0.35] | 0.09  [-0.09; 0.25] | -0.06  [-0.28; 0.18] | 0.00  [-0.12; 0.26] | 0.42***  [0.26; 0.56] | - |  |  |
| 12. State anxiety | 0.29**  [0.11; 0.45] | 0.19  [0.04; 0.35] | 0.57***  [0.42; 0.69] | -0.22*  [-0.41; 0.00] | 0.03  [-0.18; 0.24] | 0.13  [-0.08; 0.33] | 0.13  [-0.05; 0.29] | 0.00  [-0.21; 0.21] | -0.08  [-0.19; 0.17] | 0.36***  [0.19; 0.51] | 0.73***  [0.59; 0.84] | - |  |
| 13. Trait anxiety | 0.26*  [0.05; 0.46] | 0.19  [0.03; 0.37] | 0.47***  [0.32; 0.61] | -0.15  [-0.35; 0.05] | -0.02  [-0.21; 0.19] | 0.16  [-0.04; 0.33] | 0.15  [-0.04; 0.33] | -0.10  [-0.30; 0.10] | -0.02  [-0.16; 0.23] | 0.37***  [0.19; 0.53] | 0.69***  [0.55; 0.79] | 0.77***  [0.66; 0.84] | - |
| 14. Anxio-depressive symptoms^4^ | 0.33**  [0.14; 0.50] | 0.21*  [0.06; 0.37] | 0.58***  [0.45; 0.70] | -0.18  [-0.39; 0.04] | 0.04  [-0.16; 0.23] | 0.18  [-0.03; 0.36] | 0.14  [-0.05; 0.29] | -0.06  [-0.27; 0.16] | -0.04  [-0.16; 0.22] | 0.42***  [0.25-0.57] | 0.89***  [0.82-0.94] | 0.92***  [0.88; 0.95] | 0.91***  [0.87; 0.93] |

Notes. N=100. Values in the table are Pearson’s correlation estimates (r) and robust bootstrap-based, bias-corrected, accelerated 95% confidence intervals (95% BCa CI). Level of significance: *p<0.050; **p<0.010; ***p<0.001. ^1^Coded as: 0=Female, 1=Male. ^2^Coded as: 0=Vocational graduation, 1=Lack of vocational or high-school graduation. ^3^Coded as: 0=No family history, 1=Presence of family history. ^4^Composite Principal Component Analysis score based on the variables measuring depressive symptoms, state and trait anxiety
