# Supplementary material for: Externalizing personality characteristics define clinically relevant subgroups of alcohol use disorder
Source: PLoS One. 2022 Mar 18;17(3):e0265577. doi: 10.1371/journal.pone.0265577 (PMC8932598; doi:10.1371/journal.pone.0265577)
Supplement: S2 Table — Note. AIC = Akaike Information Criteria; BIC = Bayesian Information Criteria; SSA-BIC = Sample size adjusted Bayesian Information Criteria; LMR-LRT = Lo-Mendel-Rubin adjusted likelihood ratio test. 1These solutions might not be trustworthy due to local maxima (i.e., the best loglikelihood value was not replicated). (DOCX) [file pone.0265577.s002.docx]

Supplementary Table 2. Model fit indices of the different latent classes (N=99)

|  | AIC | BIC | SSA-BIC | Entropy | LMR-LRT | p | Class sample sizes |
| --- | --- | --- | --- | --- | --- | --- | --- |
| 1-class model | 854.85 | 870.42 | 851.47 | - | - | - | Class 1: N=99 (100%) |
| 2-class model | 814.76 | 840.71 | 809.13 | 0.70 | 45.61 | 0.003 | Class 1: N=46 (46.47%); Class 2: N=53 (53.54%) |
| 3-class model^1^ | 812.72 | 849.05 | 804.83 | 0.85 | 9.53 | 0.237 | Class 1: N=40 (40.40%); Class 2: N=48 (48.49%); Class 3: N=11 (11.11%) |
| 4-class model^1^ | 808.51 | 855.22 | 798.38 | 0.86 | 11.58 | 0.294 | Class 1: 15 (15.15%); Class 2: 11 (11.11%); Class 3: N=28 (28.28%)  Class 4: 45 (45.46%) |

Note. AIC = Akaike Information Criteria; BIC = Bayesian Information Criteria; SSA-BIC = Sample size adjusted Bayesian Information Criteria; LMR-LRT = Lo-Mendel-Rubin adjusted likelihood ratio test. ^1^These solutions might not be trustworthy due to local maxima (i.e., the best loglikelihood value was not replicated).
